# Preparing clinical champions for sustainable implementation of practice change within large healthcare systems

**DOI:** 10.1186/s43058-026-00873-7

**Published:** 2026-01-31

**Authors:** Sophia M. Bartels, Zenith Rai, Matthew Martel, Omonyele Adjognon, Kelly Dvorin, Charles Engel, Tamara Schult, Timothy M. Doherty, Bo Kim, Justeen Hyde

**Affiliations:** 1 US Department of Veteran Affairs, Center for Health Optimization and Implementation Research, VA Bedford Healthcare System, Bedford, USA; 2 US Department of Veteran Affairs, Center for Health Optimization and Implementation Research, VA Boston Healthcare System, Boston, USA; 3 Department of Health Law, Policy & Management, Boston University School of Public Health, Boston, USA; 4 US Department of Veteran Affairs, Seattle-Denver Center of Innovation for Value-Based and Veteran-Centered Care, VA Puget Sound Healthcare System, Seattle, USA; 5 Department of Psychiatry & Behavioral Sciences, University of Washington School of Medicine, Seattle, USA; 6 Office of Patient Centered Care & Cultural Transformation, Veterans Health Administration, Arlington, USA; 7 Department of Psychiatry, Harvard Medical School, Boston, MA USA; 8 Department of General Internal Medicine, Boston University School of Medicine, Boston, USA

**Keywords:** Clinical champions, Training, Change management, Whole health

## Abstract

**Background:**

Clinical champions can be effective for increasing uptake of evidence-based interventions. However, little is known about how to prepare them to be impactful, particularly within large healthcare systems. We present a conceptual model, grounded in the Awareness, Desire, Knowledge, Ability, Reinforcement (ADKAR®) change management framework, to guide training for clinical champions.

**Methods:**

In 2021, the U.S. Department of Veterans Affairs implemented clinical champions in primary care and mental health services to facilitate uptake of Whole Health, a person-centered holistic approach to healthcare. Our conceptual model was created through iterative team discussions about learnings from our evaluation of Whole Health clinical champion implementation. This evaluation included two rounds of interviews with clinical champions, and three rounds of a practice reflection survey (aligned with ADKAR) administered to champions.

**Results:**

Drawing on these data and ADKAR, we developed a conceptual model of how clinical champions can be supported through two complementary and sequential change management processes. The first process is related to their practice change. Clinical champions must start by gaining awareness of and interest in the new practice. They can then develop foundational knowledge and skills to enact it. Finally, they will only maintain the practice if they observe benefits of its use. Once they have progressed through the ADKAR stages in relation to the practice change, the second process they must undertake is in relation to the clinical champion role. They must first understand why clinical champions are needed and have an interest in the role. They then need training and skills for the role (e.g., overcoming barriers, mentorship). Finally, to continue the role over time they must see that champions are making a difference. Only after champions have gone through both processes can they effectively support their colleagues in progressing through the ADKAR stages to implement the change in their practice.

**Conclusions:**

Given that clinical champions are a widely used implementation strategy, this work holds promise for improving its impact on implementation and effectiveness outcomes. By supporting tailoring training to where champions are in the change management processes, our data-driven conceptual model can improve champions’ effectiveness as change agents.

**Supplementary Information:**

The online version contains supplementary material available at 10.1186/s43058-026-00873-7.

Contributions to the literature

- Little is known about how to prepare clinical champions to be effective in their roles, particularly within large healthcare systems.
- To respond to this gap, we developed a data-informed conceptual model to guide the preparation of clinical champions. Our model is grounded in interview and survey data from a partnered evaluation of implementation of clinical champions to support a new model of care within the Veterans Health Administration.
- This model includes multiple change management cycles that in combination with assessment tools can be used to tailor champion training so that they can effectively support their colleagues in adopting practice changes.

## Background

Implementation of changes or new approaches in healthcare practices among busy primary care and mental health clinicians is dependent on a variety of factors. These include clinicians’ knowledge about the rationale for change, self-efficacy and skills to undertake implementation, and motivation to adopt and change practice. One strategy to facilitate the uptake of practice changes in healthcare settings is the use of clinical champions. Clinical champions are healthcare professionals within a clinic who are responsible for providing support, advocating for, and leading implementation of a practice change [1]. Successful clinical champions are those who are passionate about the change being implemented, hold a position of respect or “informal leadership” in the clinic that can be used to influence other clinicians to adopt the practice, and who have strong mentorship and communication skills [1]. Clinical champions who possess these characteristics have greater potential to work effectively with clinicians and others to strengthen their knowledge, capacity, and interest in changes being implemented [2].

There has been research examining characteristics that make clinical champions effective, the different types of activities that champions undertake within their role, and their overall effectiveness as an implementation strategy [2–6]. Yet, little is known about the best ways to prepare clinical champions to be impactful in their roles [4]. Previous literature provides little, or no, description of how clinical champions were trained. Instead, there is often an implicit assumption that champions already have the skills and knowledge necessary to be effective in their roles, or champions are assumed to have “intrinsic qualities” that cannot be taught [4]. For example, previous conceptual models of how champions enact change have identified the importance of champion knowledge and self-efficacy, but do not explicate how these fundamentals can be developed [1, 7]. As far as we know, only one review has been published on training and education for champions. This review confirmed that there is little published about clinical champion training content, scant focus on promoting and managing champion-mediated organizational change, and no published trials that have evaluated clinical champion trainings [8]. Additionally, the findings from this review bring attention to the lack of follow-up training for champions and information around training content tailored to champions’ knowledge and practice level [4, 8].

Although identifying and preparing champions to support practice change is one of the Expert Recommendations for Implementing Change (ERIC) strategies (a well-known compilation of implementation strategies), as described above, the “preparing champions” component of this strategy requires greater focus [9]. While it may be possible within the context of clinical trials to hand-pick champions who already have the characteristics of “good clinical champions,” this is seldom feasible within large healthcare systems that have high levels of variability across provider types and sites of care. Furthermore, the assumption that healthcare professionals already have the necessary skills to be effective clinical champions is unwarranted. There is a clear need for more explicit, theory-informed guidance on how to prepare health professionals for success as clinical champions [3].

**Fig. 1:**
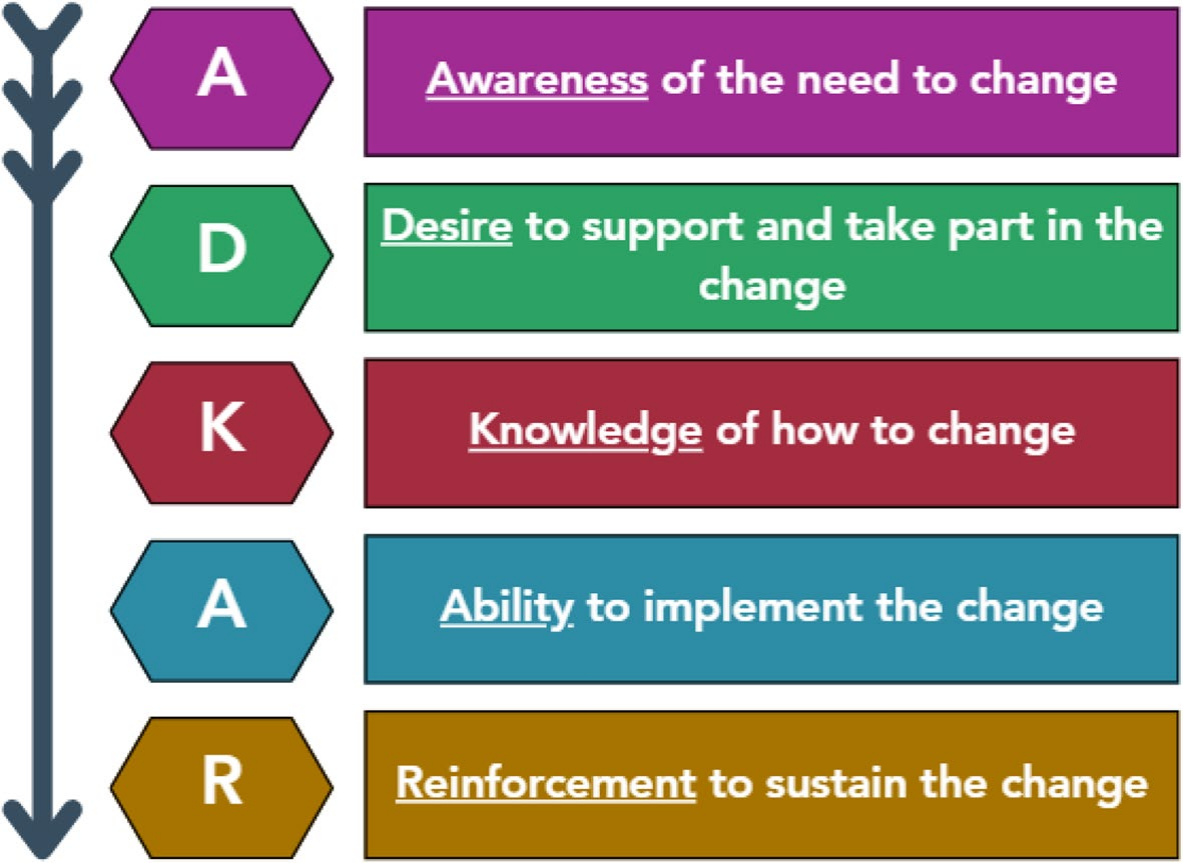
ADKAR® model

In this paper we present a conceptual model for preparing effective clinical champions. The model was developed based on our experience evaluating the use of clinical champions as part of a bundle of implementation strategies to promote uptake of a multi-component practice change within the Veterans Health Administration (VHA). Our model is grounded in a change management framework called the Awareness, Desire, Knowledge, Ability and Reinforcement (ADKAR) model® [10]. Change management is a systematic approach to dealing with transition and transformation within an organization that allows for the effective implementation of new policies, practices, and approaches to care [11]. Unlike behavior change theories focused on the individual, like the Capability, Opportunity, Motivation, and Behaviour (COM-B) model, change management is focused on large-scale organizational change [12]. ADKAR stands for the five stages of change: Awareness, Desire, Knowledge, Ability and Reinforcement. It has been used across many organizations and hospitals to support change efforts and evaluate readiness to change (see Fig. 1 for ADKAR model and definitions) [13]. We chose to focus on ADKAR, rather than other common change management frameworks like Lewin’s Change Management Model and Kotter’s Change Model, because a) it is the change management framework adopted by VHA, and b) ADKAR has a unique focus on inspiring change within large organizations (like healthcare systems) at the level of the individual, which is the ultimate goal of our clinical champion work within the VHA [14].

There is also scant literature on the role of clinical champions in supporting large system transformation initiatives that entail multiple components. We believe that a change management framework offers leaders of large system changes an approach to understanding their clinical champions’ diversity of experiences and skills. This approach can then be used to tailor training and support appropriate to different levels of awareness, desire, knowledge, ability, and reinforcement related to both the practice change and the clinical champion role. In this paper, we will first describe the clinical champion initiative that served as the context for our conceptual model. Next, we will present the qualitative and quantitative data that informed the development of our model, and finally, we will describe the creation of the model itself.

## Methods

### Setting/context

The VHA is the largest integrated healthcare system in the United States. It is comprised of 170 medical centers and 1,062 outpatient sites of care, varying in complexity and purpose [15]. VHA provides healthcare services to over nine million military Veterans living in the 50 U.S. States and Territories. Within VHA, there are multiple national program offices dedicated to establishing policies in support of high-quality healthcare services, responding to congressional mandates and inquiries, and facilitating research, training, and quality improvement efforts to assure equitable access to timely, patient-centered care [15, 16].

More than a decade ago, the Office of Patient Centered Care and Cultural Transformation (OPCC&CT) under the Office of Patient Care Services launched an ambitious effort to shift VHA’s orientation to healthcare from one that is predominately disease focused to one that is whole person focused [17–20]. The model is known as Whole Health care. Whole Health care represents a health system approach to care that is patient-centered, attentive to multiple domains of life that influence health and well-being, and focused on what matters most to patients. Whole Health care offers greater choice in health care treatments, inclusive of a range of complementary and integrative health services. It also offers a greater variety of educational and support resources (e.g., health and wellness coaching, opportunities to explore life purpose) to help Veterans discover and work towards personal health goals that may improve their overall health and well-being.

**Fig. 2:**
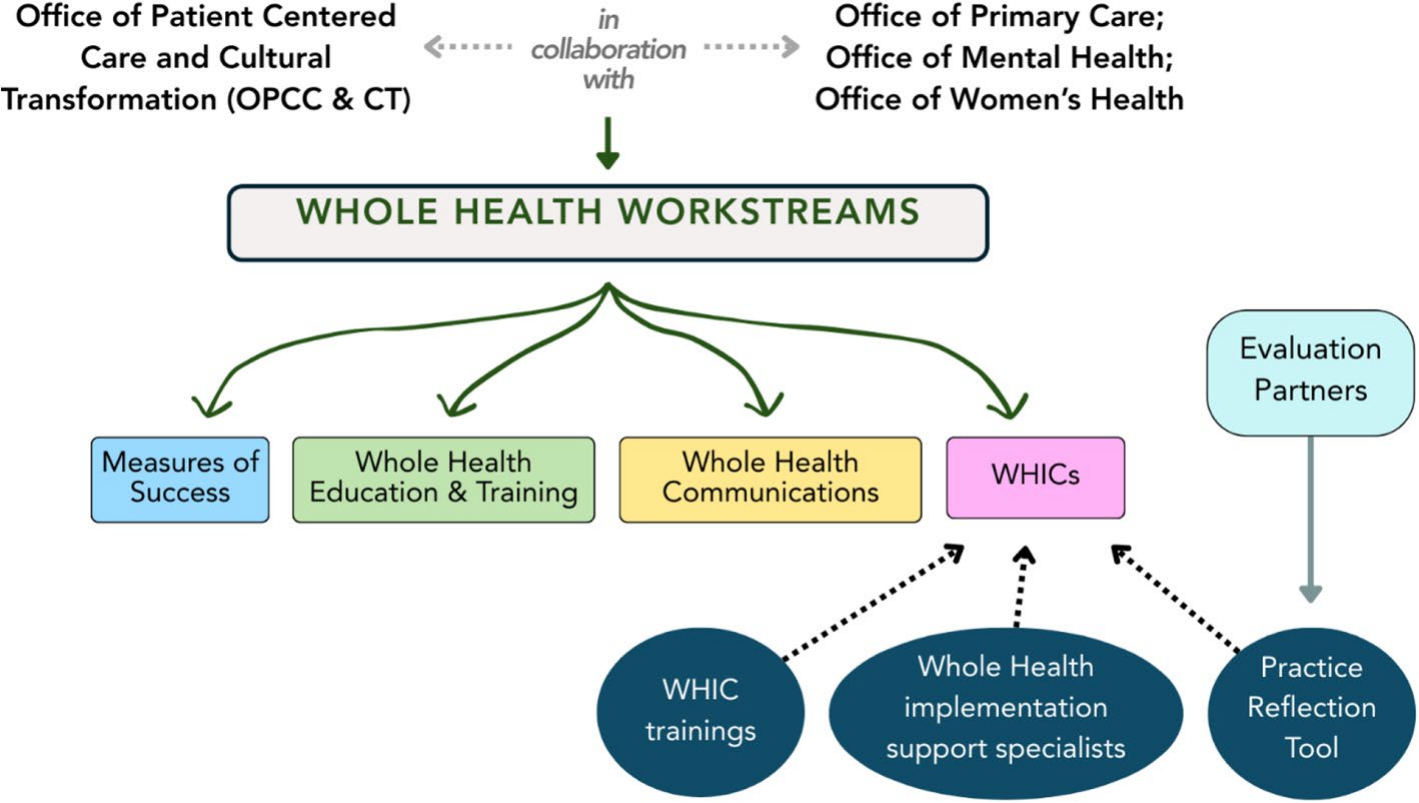
WHIC support and evaluation

Since its inception, OPCC&CT has embraced the value of operating within a Learning Health System by developing guidance for frontline employees and supporting pilot initiatives to study implementation of training, new practices, workflows, patient and provider experiences, and impacts [21–25]. Following findings from a multi-site implementation and outcome evaluation of Whole Health care in 18 medical centers [26], a larger three-year phased implementation initiative was launched in 2021 to catalyze implementation of Whole Health care in primary care and mental health services (VHA’s largest service lines). This system-wide rollout was led as a collaborative effort between OPCC&CT and two additional national program offices that oversee primary care and mental health services across VHA. The planned roll out was organized by four “workstreams,” each of which were centrally coordinated and managed by multi-disciplinary teams comprised of employees working across organizational levels, from national program office to front-line clinical services. The four workstreams included: 1) education and training for employees and leaders on Whole Health, 2) communications to support efforts to raise awareness about Whole Health and support on-going education for providers and Veterans, 3) clinical champions, called Whole Health Integration Champions (WHICs), to support adoption of practice changes within local primary care and mental health clinics, and 4) measures of success, which focused on defining and evaluating success of the change effort. Recognizing the rollout of Whole Health care as a large system change, OPCC&CT introduced and utilized the ADKAR change management framework to provide general guidance on the development of activities associated with workstreams 1–3 (education and training, communications, and WHICs) (see Fig. 2 for WHIC Support and Evaluation). Throughout the paper, we use the term “WHICs” to refer to the specific Whole Health clinical champion position within the VA, while we use the term “clinical champions” to refer to the role more broadly.

As evaluation partners for this system-wide rollout of Whole Health care, one of our priorities was the evaluation of the WHIC role. Our evaluation was intended for quality improvement purposes. We collected data to understand how the WHIC role was being implemented across facilities, facilitators and barriers to performing WHIC responsibilities, promising practices for the role, and recommendations to support WHIC effectiveness. Our conceptual model for clinical champion preparation was developed based on our experiences throughout multiple data collection, analysis, and dissemination cycles as WHICs were implemented in three consecutive waves across the VHA system. The Standards for Quality Improvement Reporting Excellence guidelines were used as a framework for reporting this manuscript (Additional File 1) [27].

### Formative data collection

**Fig. 3:**
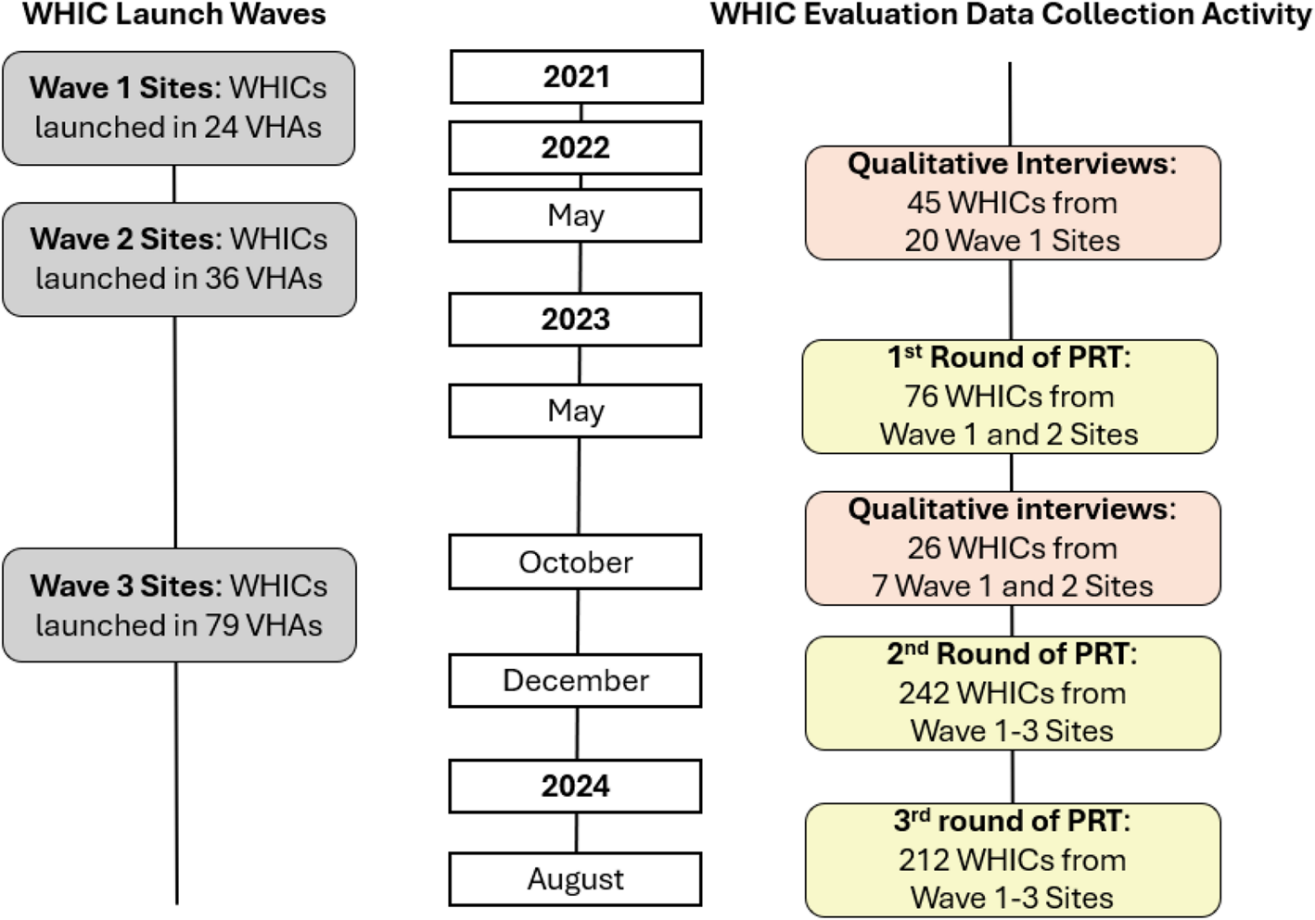
WHIC data collection timeline

Our quality improvement evaluation entailed iterative cycles of qualitative and quantitative data collection with WHICs in primary care and mental health services. This data collection included: 1) two rounds of semi-structured interviews with WHICs to learn about how Whole Health implementation was going at their site and barriers and facilitators to success in the WHIC role, and; 2) three rounds of administration of a survey-based evaluation and self-reflection tool that our team developed (called the Practice Reflection Tool) that provided quantitative data on how WHICs were doing across the ADKAR domains (see Fig. 3 for data collection timeline).

Data collection started with semi-structured interviews with wave 1 WHICs. For these first-round interviews, all WHICs in the 24 wave 1 sites were invited to participate in an interview. The interview guide was developed by members of our research team (with input from OPCC&CT partners) and focused on topics including approach to the WHIC role and implementation facilitators and barriers, including coordination with the facility Whole Health leadership, support from service-line managers, circumstances within practice settings, and clinical team interest. Analysis of the first wave of interviews led to insights regarding high variability in areas aligned with the ADKAR framework (described more fully in the “Results” section).

Several participants also noted during interviews that it would be good to have milestones that WHICs could use to inform both what they needed to learn and actions they could take in their own clinical practice and in their role as a WHIC. Following wave 1 interviews, our team proposed to OPCC&CT the development of a tool that was recommended in the interviews to increase awareness of personal and role-specific desire, knowledge, ability, and reinforcement for Whole Health adoption. We worked with the WHIC taskforce to develop this tool, which became known as the Practice Reflection Tool (PRT). Following the first administration of the PRT, we conducted a second round of interviews with Wave 1 and 2 WHICs, using essentially the same interview guide as the round 1 interviews, but exploring their ongoing experiences and perspectives. For the second-round interviews, given the greater number of sites and WHICs, we undertook a site selection process. Our criteria for selecting sites included that they had high participation in the pilot PRT survey (conducted May 2023) and higher than average scores across PRT survey domains. Our goal was to select sites with WHICs who perceived themselves to have knowledge of Whole Health and the WHIC role. From these criteria, we selected eight sites for interviews (seven sites agreed to participate) and invited all WHICs and Whole Health leads within these sites for an interview. We then re-administered the PRT at two additional time points with WHICs from Waves 1–3.

The PRT questions were informed by 1) themes that emerged from the qualitative interviews, 2) the ADKAR domains, and 3) the Designation Framework for Whole Health Implementation [28], a guide to Whole Health that describes activities occurring in each phase of the “Whole Health Implementation Journey.” It included the following sections: 1) knowledge and practice of Whole Health at the individual clinician level, 2) their understanding of and practices enacted as a WHIC, 3) their engagement in self-care practices, and 4) contextual factors that may have impacted success in the role (see Supplementary Material 1 for example PRT questions). WHICs rated questions in these domains on 5-point Likert scales (e.g., 1 = little knowledge, 5 = extensive knowledge). Before full implementation, the PRT was pilot tested with a sample of WHICs and Whole Health leaders (round 1 administration). The PRT questions were administered to the WHICs electronically and remained largely consistent across the three rounds, apart from the addition of a few questions related to new Whole Health tools that had been developed.

### Formative data analysis

PRT data was analyzed using descriptive statistics, including for each PRT round calculating average scores for each PRT question. For dissemination to the clinics and OPCC&CT, we calculated the percentage of respondents who reported “high levels” (i.e., a “4” or a “5”) for each PRT question (e.g., “high knowledge”). We also conducted independent t-tests in Stata to compare changes in PRT responses over time (between PRT rounds). These results were aggregated at the clinic and region-level and shared in the form of written reports that highlighted potential areas of focus for future training activities.

For the qualitative data, prior to conceptual model development members of our team with qualitative expertise (JH, KD, OA, ZR, MM) had conducted a rapid qualitative analysis following each round of interviews [29]. The analysis included team members summarizing key information from each interview using structured templates in word (round 1 interviews) and Excel (round 2 interviews) organized by main interview domains (e.g., prior experience/training in Whole Health, understanding of the WHIC role, etc.). To inform conceptual model development, SB and JH conducted a targeted secondary analysis of the interview data, reviewing the summaries from both interview waves and using a matrix to map the data and illustrative quotes to ADKAR domains. We then triangulated the interview and PRT survey data through side-by-side comparison of the qualitative and quantitative findings by ADKAR domain to provide us greater confidence in our findings, with the qualitative data also helping to provide context and possible explanations for the quantitative findings [30]. In our results section, we use parentheses following quotes from our secondary qualitative analysis to denote WHIC role type, site, and participant number (e.g., “PC 02–04” indicates this was a primary care WHIC from site 2 with participant number 4).

### Conceptual model development

**Fig. 4:**
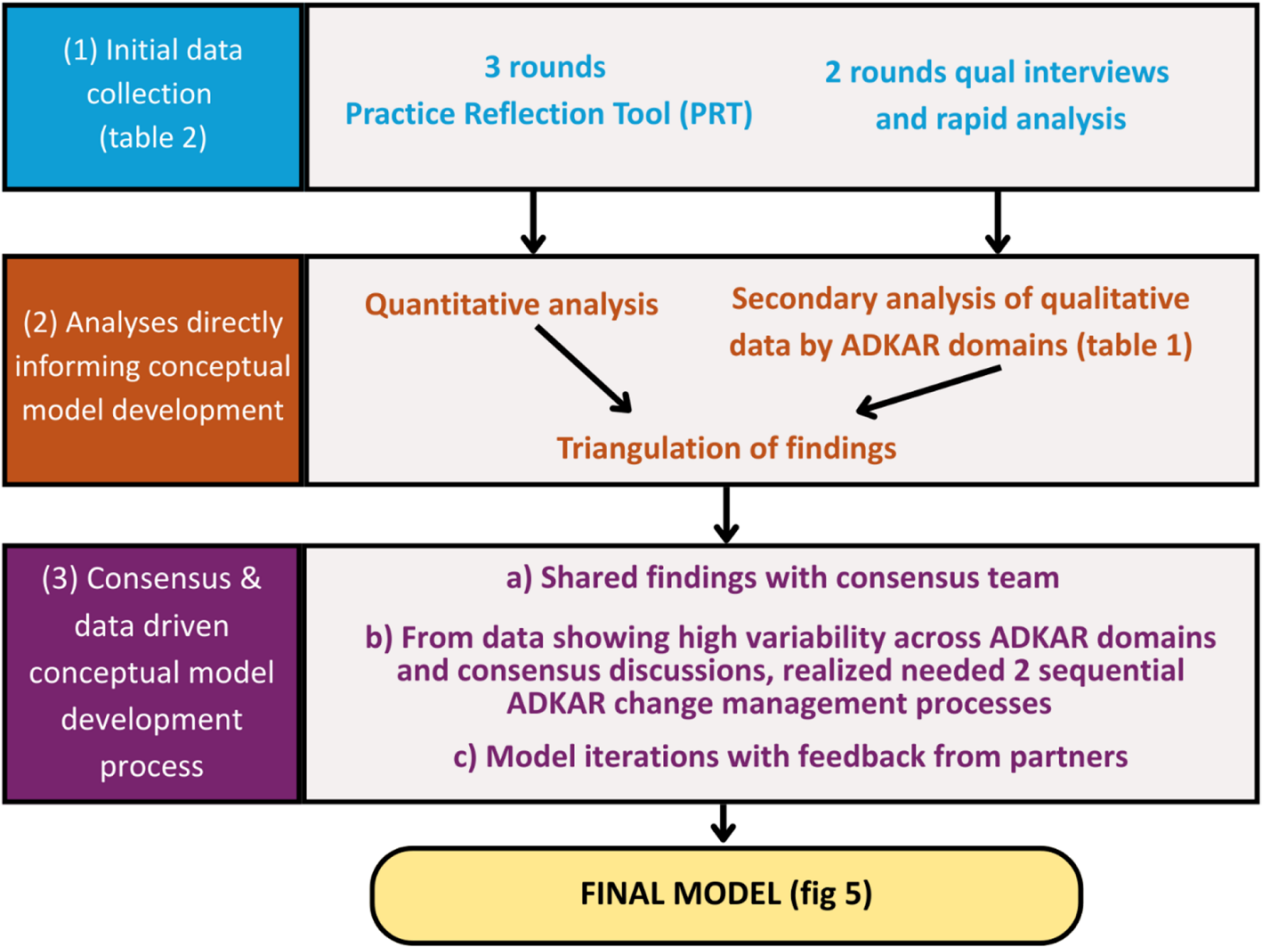
Data and consensus-driven conceptual model development process

Following analysis of the interviews and surveys, our team used an adaptation of an iterative consensus group approach [31], informed by this data and the ADKAR framework, to create our conceptual model. Members of our team had previously developed this approach, based on established decision-making methods, to gather and synthesize data and expert opinions. Team members involved in our consensus process included implementation scientists, anthropologists, systems scientists, clinician researchers, and representatives from OPCC&CT. Our adapted consensus process was done in 7 steps: 1) team held an initial brainstorming of model components (e.g., ADKAR processes for the clinical champion’s personal practice adoption and the clinical champion’s role adoption) and their relationships based on findings from the interviews and PRT surveys; 2) authors Bartels and Hyde reviewed and consolidated the brainstormed components and relationships into a preliminary draft model, including taking each ADKAR domain and using our data to modify the definition to be applicable to the clinical champion’s personal practice adoption (1st phase in model) and then to the clinical champion’s role adoption (2nd phase in model); 3) team provided input on how to clarify and improve the draft model, holding structured consensus-reaching discussions to resolve any discrepant suggestions; 4) authors Bartels, Hyde, and Rai incorporated team member suggestions to update the model; 5) model was shared with OPCC&CT partners for feedback; 6) authors Bartels, Hyde, and Rai incorporated OPCC&CT partners’ suggestions to update the model; 7) team iterated through Steps 3 and 4 to refine the model until there were no more suggestions to discuss and incorporate (see Fig. 4).

## Results

### Formative qualitative findings informing the conceptual model

In 2021, WHICs were trained and launched in the first wave of 24 VHA medical facilities; in May 2022 qualitative interviews were conducted with a sample of 45 WHICs from 20 of these sites. Among the most critical findings, we learned that WHICs were not operating with the same levels of awareness of, desire for, knowledge of, and experience with providing Whole Health care. Some WHICs were “voluntold” to serve in the WHIC role and others volunteered. Some had participated in multiple local and national trainings on Whole Health care while others reported that the WHIC orientation training was their first exposure to Whole Health. Additionally, a number of WHICs lacked clarity about the purpose of the clinical champion role and were unclear about the activities they were supposed to be doing to carry it out. This feedback was provided to OPCC&CT national leads, who quickly expanded training options for the second wave of WHIC sites (n = 36 sites) to include a basic training on Whole Health care for “novices” in addition to the revised two-hour training providing an overview of the WHIC role.

Prior to the launch of wave 3 sites (n = 79 sites), we conducted a second round of interviews in October 2023 with a sub-sample of 26 WHICs and Whole Health leads from wave 1 and 2 sites. From these interviews we learned additional lessons that shaped our conceptual model. Most, but not all, WHICs understood the rationale for Whole Health care and expressed some desire for making this shift in care: “*It really is the only way I practice. It just – I believe it so strongly”* (PC 02–04). However, participants had highly variable knowledge and personal practice related to Whole Health. For some, Whole Health was well-integrated into their practice: “*I use it explicitly at the beginning of therapy with each of the patients”* (MH 01–02). Others did not have time to make it a priority: “*I’m the code orange RN and I have a Veteran in my room or I’ve got this fire I’m putting out for one of my mental health patients and then that Whole Health meeting becomes the least important*” (MH 02–02). Additionally, WHICs tended to be early in their awareness of what the WHIC role entailed: “*I knew quite a bit about Whole Health but as far as my exact role I just kind of in the beginning sat back and – and watched and I wasn’t 100 percent sure what my role was”* (MH 04–03). Across the sample we found variability in WHICs’ desire to undertake the role and a wide range of capacity and skills for the role. Some participants had no time for the role: “*I still haven’t been mapped for it. So, I haven’t got any protected time for it*” (PC_06-04). While others felt fully prepared for the role: “*I mean I’m a change agent. I’ve got every tool in my toolbox of how to help with change and how to have these difficult conversations with people who are sticks in the mud, and all of that.”* (PC 02–04).

Although WHICs had been introduced to Whole Health care as a change process and provided with opportunities to learn about the ADKAR change framework, these interviews shed light on the need for WHIC workstream leaders (described above) to consider WHIC preparation through a change management lens. In particular, they highlighted how varied champions were in terms of their understanding of Whole Health care and their implementation approach in their own practice with patients. Interviews also helped elucidate WHICs’ understanding of and engagement in the WHIC role. For example, providing a training on the WHIC role without WHICs first having a good grasp and use of Whole Health approaches in their own personal practice was akin to putting the proverbial “cart before the horse.” (see Table 1 for qualitative results summaries and full illustrative quotes by ADKAR domains). However, we also recognized that our qualitative interviews were only conducted with a subsample of WHICs from a small number of VHA facilities. The extent to which these early experiences in the role were similar across the larger sample of WHICs was unknown, which was the impetus for the development of the Practice Reflection Tool (described more in “Methods”).

**Table 1:** Qualitative results summaries and full illustrative quotes by ADKAR domains

ADKAR Domain	Summary of Issues/Variability	Illustrative Examples from WHICs
Clinical Champion’s Practice Adoption		
Awareness	Some WHICs saw Whole Health as necessary in their role and even that their “position requires it.” Others were not convinced that it was different than the care they already provided to patients, which then made it harder to carry out their WHIC role	Sees Whole Health as necessary in her role: *I think my position requires it. I have to look at any individual holistically to try to achieve permanent housing. A lot of the barriers that make it difficult for them to maintain housing has to do with like their entire being. Right. It's never just one thing. It's never just the substances. It's never just the trauma. It's never just finances. It's everything. So I think it requires a like certain holistic, humanistic approach to even start doing any of the work that I do. So I figured I'm already doing it. Oftentimes, my clients don't have the same goals as me. They think that what I want for them is harmful in some instances. And sometimes I have to ask them, what's most important to you right now? And a lot of times, the answers aren't really in alignment with them keeping housing, I think it helps build rapport, especially our population specifically, they're really disengaged from the VA system, and so they tend to have a lot of experiences that don't feel welcoming or warm or meet their needs. So because I'm out there with them, I have to take a different approach. (SW 12–12)* Not convinced that Whole Health is different than regular care they provide: *I do have my own questions that come up when I say how is this different than what I would do without the label Whole Health? How is this different? And I think it is a little bit more expansive than what I would consider. At the same time, it feels like this is what we do for mental health. We don't just look at symptoms, we look at overall functioning. We look at – I don't think I would say it the way I'm saying it these days, but we go at it by helping people to do what's most important to them. So there's a little bit of a feeling of we're expanding but repackaging at the same time. So I feel like in that sense, that either we're overstating things in terms of the transformation that Whole Health is or there's something that I'm missing. And I suspect it may be a little bit of both that I don't know. If I were to run into a colleague who wasn't concerned about the wellness of the person as opposed to treating the difficulties that they come in with, I would wonder how they got through the interview process because I see that as the overall model. (MH 01–03)*
Desire	Some WHICs were really interested in and motivated to implement Whole Health. Others were not convinced that it was different than the care they already provided to patients, which then made it harder to carry out their WHIC role	Motivated and interested in implementing Whole Health: *You just have to have a crew that is motivated and resonates with the idea of Whole Health, and I've been lucky enough to be on a crew that they actually like it. So, they have been very, very enthusiastic also. So, it's not only that is a physician thing, you know, it's a group thing, right…So, we have had very good, very good experiences so far with – with these – with the Whole Health concept. (PC-04–05)* Not convinced that Whole Health is different than regular care they provide: *I do have my own questions that come up when I say how is this different than what I would do without the label Whole Health? How is this different? And I think it is a little bit more expansive than what I would consider. At the same time, it feels like this is what we do for mental health. We don't just look at symptoms, we look at overall functioning. We look at – I don't think I would say it the way I'm saying it these days, but we go at it by helping people to do what's most important to them. So there's a little bit of a feeling of we're expanding but repackaging at the same time. So I feel like in that sense, that either we're overstating things in terms of the transformation that Whole Health is or there's something that I'm missing. And I suspect it may be a little bit of both that I don't know. If I were to run into a colleague who wasn't concerned about the wellness of the person as opposed to treating the difficulties that they come in with, I would wonder how they got through the interview process because I see that as the overall model. (MH 01–03)*
Knowledge	There was variability in WHICs’ knowledge about Whole Health; some were very familiar with it, whereas others only had “a little bit of knowledge” coming into the WHIC role	Previous Whole Health training: *I’m lucky that most of the champions that I have, have been around the VA [REDACTED] primary care for a few years. And they’re very familiar with, you know, patient centered care and setting goals and they’ve taken TEACH and Motivational Interviewing and things like that. So, they – they’re pretty geared. (PC-01–01)* Little previous Whole Health knowledge*: Again, it [serving as a champion] was just something that was offered, and I had a little bit of knowledge of Whole Health. Not a lot, but it sounded interesting enough to give it a try and see where it can go. So I think it was worth it, and it's still worth it. I'd like to see it through to where it could be fully implemented and see what it looks like. (MH 5–01)*
Ability	There was wide variability in how much WHICs had integrated Whole Health into their own practices, from some WHICs integrating Whole Health into appointments with every patient, to others feeling that with an overall lack of time and other responsibilities Whole Health became the “least important” activity	Whole Health is well-integrated into their practice: *I use it explicitly at the beginning of therapy with each of the patients. The way I do that is I use the Personal Health Inventory to help get to values and what's most important for the Veterans so that we can identify what the presenting issue is and look at how as they make progress, it impacts those most significant areas to them. So between Whole Health and being trained in acceptance and commitment therapy, I see the importance of values. So prioritizing what's most important to you and how is what brings you in getting in the way of you realizing those values and putting front and center what's most important to you. (MH 01–02)* Consciously working to use Whole Health in their practice: *I gotta start doing this myself, and how easy is this? So I started trying to implement it in my practice, and that took some time to be able to do and remember to do and all the figures, and then that was how I could be able to go to the BHIP team meetings, especially team meetings, and all along in being able to say, hey, let me talk to you about some of my experiences. (MH_12_01)* Lack of time to carry out Whole Health activities: *So, there’s lots of stuff going on that just – just isn’t really functioning very well and I think the Whole Health – the Whole Health portion of it is missing out because we don’t have that designated person and I haven’t been able to be that fulltime designated person that I would like to be…I have these meetings and the Whole Health meetings will show up and but I’ve got two other meetings I’m supposed to go to but then I’m the code orange RN and I have a Veteran in my room or I’ve got this fire I’m putting out for one of my mental health patients and then that Whole Health meeting becomes the least important*. (MH 02–02)
Reinforcement	Some WHICs even used and saw the benefit of Whole Health principles in their own lives; other WHICs found that Whole Health was not appropriate for certain high-needs patients and did not use it with them	Whole Health has been positive in their own life: *I have to say that the Whole Health process and the Whole Health – the whole thing about it helped me in my own life just from learning it and hearing it from other people. (MH-02–02)* Finds that Whole Health is not appropriate for some high-needs patients: *I have to be very very careful. …I would feel as if you were disrespecting the patient if they came in here and they literally don’t have a coat, they’re asking for bottled water… I know I would never do that. If I focused on what I wanted to talk about as opposed to an obvious basic need. Here’s an example…In my drawer here is a pillow. I have a blanket but when people come here, and some of them have not slept for days, and so this is how basic this is and people are shivering, they’re cold. I had a man come here yesterday, “Where can I go to take a shower?” And for me then to say, “Hey, I know life is difficult, but have you ever thought about doing this?” I – I cannot say that to somebody…[if] I saw a coworker focused on that type of stuff when it’s obvious this is for a person who is in dire need, I would have to go say this is not what we’re – this is not the goal right now. (MH 5–02)*
Clinical Champion’s Role Adoption		
Awareness	There was variability in WHICs’ understanding of the need for champions, with some seeing the importance of having effective “change agents” and others not understanding what the purpose of the WHIC role was	Recognizes need for effective WHICs: *You have to be somebody who is a change agent. You have to be someone who is real – realize that you’re going to come against some like hands, you know hands up and make a little bit – you have to be a real ally, you know, and an ally isn’t silent. (PC 02–04)* Did not understand what the WHIC role was*: I knew quite a bit about Whole Health but as far as my exact role I just kind of in the beginning sat back and – and watched and I wasn’t 100 percent sure what my role was. (MH 04–03)*
Desire	There was variability in WHICs’ original desire to take on the WHIC role; some happily volunteered while others were “voluntold.”	Volunteered: *Whole Health is extremely important, okay. We’re not just a physical entity. We’re a product of all our parts, and that’s why when I saw this I thought, well this is great because I try to convey that to people every day… So, I volunteer for that. (MH 5–02)* Voluntold: O*ur nursing director at the [REDACTED] VA…You know, she personally reached out and said, ‘I’m so glad you’re at the – you are at the annex, I need you. And I need you to be the WHIC over there and I want you to start, you know, having slots where you can see Veterans or do presentations to our’ – I don’t know what type of presentations, if they wanted it to be more of our sub therapy, to talk to them. But then we had a massive exodus of staff members and so that would’ve probably been about a year ago that she reached out to me to ask me. So, I’ve been participating in the meetings and everything. (MH 02–02)*
Knowledge	Some WHICs appeared to not have clear knowledge about what they were supposed to be doing in the WHIC role, which made it so they were not effective in their role. Others really appreciated the WHIC trainings they had received and felt they supported them well in their role	Limited Champion role knowledge: *I was just going to say that's probably been the biggest frustration, because I came into it a year ago, you know, like, what can I do? How can I prepare? And I know that I was coming in guns blazing, ready to learn, and they were like, okay, well, you know, [NAME] said, go learn as much as you can about Whole Health. There's a ton of resources in TMS. We have the conference that will be coming up. That'll explain everything, and you'll be clear after that. I was like, clear as mud after that. I had no clue what I was doing, and I did dive into the classes, but it’s kind of – it’s put me in a position now in my current role where I've been trying to put this time aside and figure out my role with Whole Health. But now I'm in a position in my clinic where it has been a year. So my management is saying, well, it's been a year. What has been going on? Because I don't have results. And it's hard to explain to somebody that when you don't have direction, there's no results to follow. And I am finding it very frustrating… (MH-04–02)* Greater WHIC role training: *As we mature within the WHIC model and continue developing skills and receive support from the VA in the form of trainings, you know, that's something that we like a lot, the trainings, because it gives us the knowledge and help us to put things in perspective that reflects our values and our mission. So, I think that is something very important for me as a WHIC to have access to those trainings so that way I can continue evolving and continue the education and sharing it with my colleagues as well (PC-04–05)*
Ability	For some WHICs, it was clear that they knew what their role was and were conducting a variety of WHIC practices. Others had little training and preparation for their role and weren’t sure what they were supposed to be doing in relation to it	Little preparation/training for WHIC role*: One thing I was really unclear about was the training. [name] had mentioned that just yesterday about, you need to get together with the other WHICs. And this is what I was saying about the other WHICs. I'm not 100% sure what that means, but – and decide on training dates. And that's when I was asking her, I must be completely in the dark, or I have missed something, or I don't want to say I know nothing about what you're talking about, but I know nothing about what she's talking…it’s kind of changed my feeling of Whole Health, unfortunately, because I started off very excited and now I'm in a position where I'm daily justifying, yet I've been trying to find out all along how to do this. It's frustrating for me. (MH-04–02)* Demonstrates use of a number of WHIC practices: *I introduce myself. I let them know that I’m their WHIC representative, and I try to not overwhelm people. I try to do my best to provide them with, like, the simplest way to integrate any Whole Health measures and to document them. …I usually kinda show them and remind them because by the time they’ve – a few weeks or a month or a couple of months have passed since the seminar, either people are using it or they’re not. And if they’re not, they’re gonna want an easy, quick refresher as opposed to, like, a deep dive because then that will just seem overwhelming…Sometimes we’ll give feedback to the clinics to let them know, “Hey, we see that you guys are doing this. Keep this up.” That way, they have some reinforcement to know that they’re being seen as opposed to them clicking buttons and charting it but then never really knowing if it’s counting for anything. So, we try to reaffirm that, “Yes, like, we’re seeing that. You’re getting credit for it. Keep up the good work,” and that hopefully reinforces that.* (MH_07_01)
Reinforcement	There was wide variability in leadership support for WHICs’ efforts, which impacted their ability to carry out the WHIC role; some WHICs found the role to be a burden and a negative influence on their life	Leadership supports WHICs’ efforts: *Hence, what I already mentioned is a lot easier if somebody’s already within the clinic advocating for that – that’s probably easier. So, I think that’s been – not to say that people have been, like, difficult with it or that we’ve had trouble, but it’s been far more effective, like I mentioned, if the director or heads of the clinics have also reinforced that or kind of welcomed us in to do that. (MH 07_01)* Lack of leadership support of WHICs’ efforts*: And we have a staff meeting every month and I’m supposed to give an update and it’s often, you know, we’ll have like an hour – like 40, 25, 35, 40 min training on something for nurses on something that really doesn’t have anything to do with the rest of – much to do with the rest of the team, but we all – you know we participate. And when it gets to Whole Health and she’ll say, okay, you’ve got one minute or 30 s. I was like, um. So that is frustrating. That’s still happening. And I do think, you know, they’ll say things like well this Whole Health stuff, you know, it’s you can do this, these trainings that [NAME] sent out, but they’re really on your time. And I had to correct her, and I said no the VA wants you to participate in these. They’re – we’re being – they’re counting how many people are trained. They’re holding us accountable. This is not a on your free time. And then one of the social workers said, well but my – we’re not given – it’s not part of our day-to-day job. And I was like, well it is, but you only can do what you can do.” (PC-02–04)* Negative reinforcement: *I've been so back and forth, back and forth on thinking I'm doing the right thing or trying not to bother people who are busy and just to kind of be a year later actually worse off than when I started. And it's like, to me, it's so backwards because it's the opposite of Whole Health and what we're supposed to be doing. And I'm stressed out. I'm doing this on my off time. I go home and I'm like, okay, I don't have time to do it now, so let me do it when I'm at home. And it's a burden. (MH-04–02)*

### Formative quantitative findings informing the conceptual model

The first round of the Practice Reflection Tool took place in May 2023 with WHICs from wave 1 and 2 sites. We found that (as in the qualitative interviews) WHICs had variability in their Whole Health knowledge and practice. Additionally, they had a greater understanding of the foundational elements of Whole Health than specifics on available Whole Health services within the VHA (Supplementary Material 2). They also lacked clarity about the expectations of the WHIC role and resources to support it. These findings reinforced the need for more intensive and differentiated training for WHICs based on where they fell in the change management process. The second round of the Practice Reflection Tool was administered in December 2023 and included new WHICs from wave 3 sites. Findings from the second round of surveys demonstrated that WHICs had similar knowledge of Whole Health and implementation of Whole Health in personal practice to May 2023 (Supplementary Material 2). Additionally, they continued to have limited knowledge about expectations of the WHIC role, reported that they were still “just getting started” or were very early in their clinical WHIC role (Supplementary Material 3). Although they may have engaged in more WHIC activities (e.g., demonstrating how to change the conversation to explore what matters most to the Veteran), their confidence in conducting these activities remained low.

Finally, the third round of the Practice Reflection Tool was administered in August 2024. One question of interest at this point was how effective the iterative rounds of training and regional implementation support (described in Table 2) were in moving WHICs along the change management process and where WHICs still needed additional support. Across the Practice Reflection Tool domains, we saw improvements between 2023 and 2024, a signal that the regional implementation specialist support may have been effective in some areas of the change management process. Statistically significant improvements (*p* < 0.01) were found in nearly all items related to WHICs’ knowledge of Whole Health (Supplementary Material 2) and half of items related to their understanding of and expectations for the WHIC role (Supplementary Material 3). We saw slight improvements on most items related to personal use of Whole Health practices and engagement in WHIC activities, but the majority of these changes were not statistically significant. Based on these results, our feedback to our partners at OPCC&CT was that while many of the WHICs, through their training, had been effectively moved through the early change management process stages, they required more training related to incorporating Whole Health into their own clinical practice. Additionally, they needed guidance on specific activities to promote their ability to locally support the uptake of Whole Health within their clinic (see implications of the formative data on OPCC&CT WHIC training and support in Table 2).

**Table 2:** Impact of formative data on OPCC&CT WHIC training & support

	**Impact of Results**
Qualitative Findings	• OPCC&CT partners used interview findings to revamp WHIC orientation and create guidance for WHICs for their role based on their familiarity with Whole Health (novice, moderate, expert) • Interview data led to development of Practice Reflection Tool
Quantitative Findings	• First round of PRT: OPCC&CT partners used PRT results to revise orientation for WHICs in wave 3 sites • Second round of PRT: Informed by PRT results, OPCC&CT leveraged expertise of their four regional Whole Health implementation support specialists to provide more in-depth trainings and tailored support to WHICs at a regional level ◦ Approach implementation specialists took to support varied by region ◦ Generally focused on more frequent skill-building trainings (with some grounding trainings in ADKAR) and brainstorming solutions to Whole Health implementation barriers, such as lack of time for WHIC role (a common challenge across regions)

Collectively, our qualitative interview and PRT response data helped us recognize: 1) that within large healthcare systems, assumptions should not be made about clinical champions’ knowledge and capacity related to the target practice change and clinical champion role and 2) that multiple change processes are needed to develop effective clinical champions (see Fig. 3). Together, the results from our evaluation of the WHIC role implementation and our teams’ learnings from across these data collection cycles supported our development of the ADKAR-informed Conceptual Model to Prepare Clinical Champions for Facilitating Systems Change model, which is described below.

### The ADKAR-informed conceptual model to prepare clinical champions for facilitating systems change

**Fig. 5:**
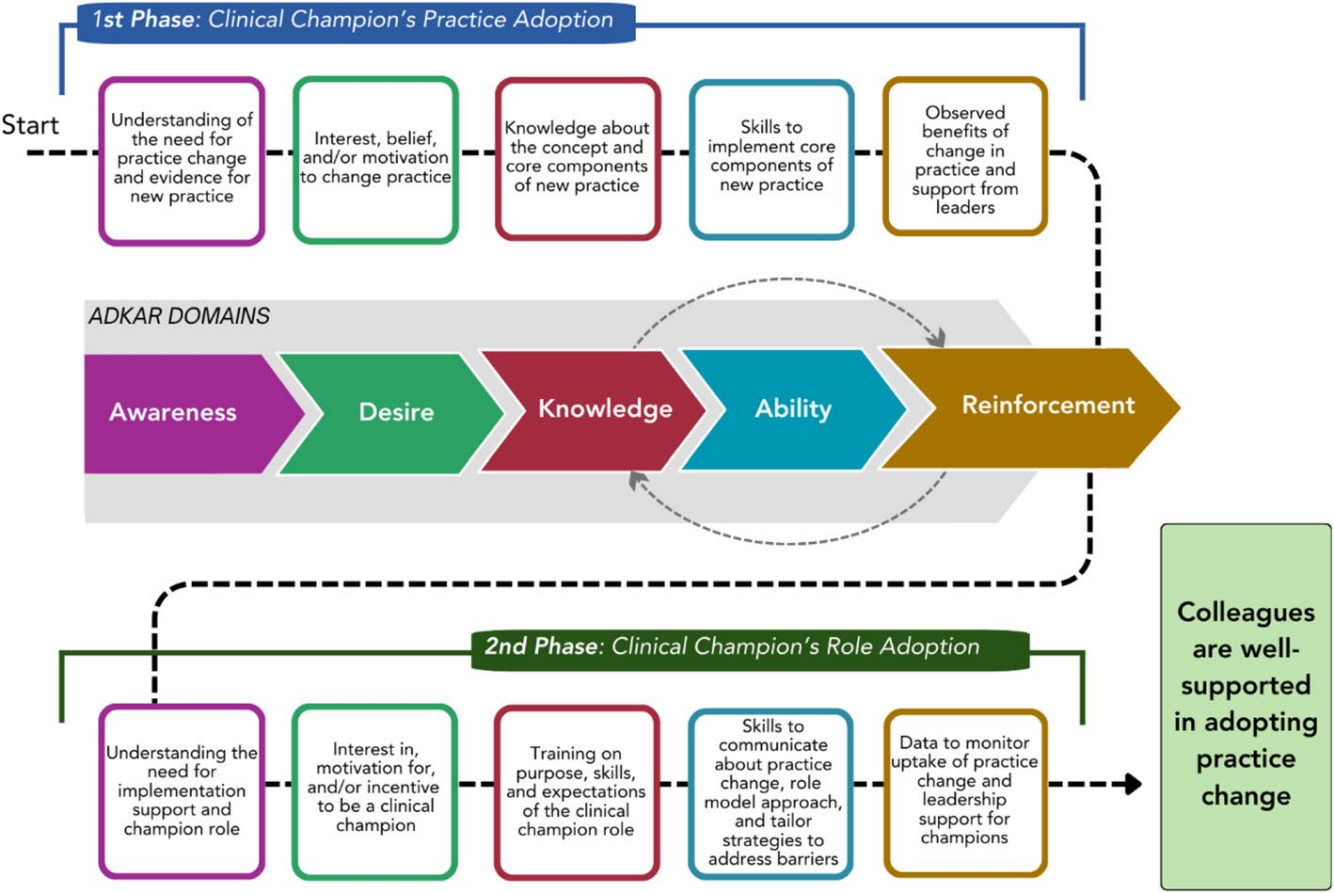
ADKAR-informed® conceptual model to prepare clinical champions for facilitating systems change

Two phases of the change management process constitute our conceptual model. The first phase is related to the clinical champion’s adoption of the practice change, while the second phase is related to the clinical champion’s adoption of the clinical champion role. The phases are sequential, and both follow the ADKAR domains (see Fig. 5 for conceptual model).

The first phase of the change management process is related to the evidence-based practice that is being implemented and clinical champions’ adoption of the practice change. Clinical champions must have a clear understanding that there is a need for a change in practice and an awareness that there are evidence-based practices that might work better than what they are currently doing (1st Phase: Awareness). Once they realize that there is a need for change, they must then be interested in and motivated to make this change within their current practice (1st Phase: Desire). Next, they need to be trained on the specifics of, and skills required to implement the new practice (1st Phase: Knowledge), so that they have both the knowledge of the practice’s “core components” and ability to carry it out (1st Phase: Ability). Finally, they need reinforcement that what they are doing is making a difference (through data and/or observed changes in their own practice) as well as support from leadership (1st Phase: Reinforcement). This support could come in the form of periodic reminders about the importance of implementing the new practice and periodic refresher trainings that reinforce champions’ knowledge and skills about the practice (among other strategies). Attention to all of these steps is needed so that a clinical champion has a strong foundation in the new practice required to be a role model and instructor for their peers.

The second phase of the change management process is related to the clinician’s adoption of the clinical champion role. Clinical champions require training both in the new clinical practice (first phase) and in how to effectively influence their peers to adopt the practice and overcome implementation barriers (second phase). In this second change management process, designated champions need to be given knowledge about and rationale for the need for implementation support (i.e., the clinical champion role) for the practice change (2nd Phase: Awareness). This understanding is important for clinicians to want to take on the clinical champion role (2nd Phase: Desire). Next, clinical champions need to be trained in the “core components” of the clinical champion role, including training on the purpose, expectations, and specific skills needed to be successful in their role (2nd Phase: Knowledge). These trainings should include hard skills, like training in educating others about the new practice, tailoring implementation support activities, monitoring ongoing use of the practice, and overcoming barriers to implementation (2nd Phase: Ability). They may also need training in “softer” skills, like communication, mentorship, and leadership [1, 7]. Finally, champions will need ongoing reinforcement related to the clinical champion role, which could take the form of periodic clinical champion community of practice calls to share best practices and lessons learned, data to monitor uptake of the practice change, and support from leadership to maintain the new practice (among others) (2nd Phase: Reinforcement).

Having moved through both change management processes, clinical champions will then be equipped to ensure that their colleagues are well-supported in adopting the practice change. Once clinical champions have gone through the change management processes themselves, related to the new clinical practice and the champion role, they will be more effective in supporting their peers in gaining awareness, desire, knowledge, ability and reinforcement in implementing the new clinical practice. In this way, our conceptual model is sequential, with each change management process building upon the other. In addition, the conceptual model is self-reinforcing because by providing their peers with training and skills in implementing a new clinical practice (Reinforcement), this can improve clinical champions’ own knowledge and abilities in delivering the practice themselves (represented by dotted gray arrows in the conceptual model in Fig. 5).

## Discussion

In this article we offered a conceptual framework to guide the preparation and practice of clinical champion roles in large healthcare systems. The conceptual framework was informed by empirical data that highlighted multiple change processes that clinical champions need to work through to be effective in their role. We found from our analysis of interview and survey data that in large healthcare systems, clinical champions require tailored training to be effective, given that their previous training in the practice change and in making change in an organization may vary significantly. We then presented the data-driven, multilevel, ADKAR-informed Conceptual Model to Prepare Clinical Champions for Facilitating Systems Change.

While ADKAR has been used in a variety of healthcare contexts to support practice change (e.g., supporting staffing changes during the COVID-19 pandemic), as far as we know, this is the first time an ADKAR-informed model has been developed to guide training and preparation for clinical champions in large healthcare systems [13, 32]. This study highlights the value of using a change management framework to support training for clinical champions. ADKAR breaks apart the steps needed to make large-scale and sustainable change within an organization, which is the ultimate goal of the clinical champion strategy. Specifically, ADKAR endorses the need to take note of where each champion is within their change management process (related to both the practice change and champion role) to effectively tailor training. ADKAR also informed our conclusion of the need to provide clinical champions with training to move them from one stage of change to the next. A lack of appropriate clinical champion preparation and assessment will limit champions’ impact on practice change and may contribute to some of the mixed results in the literature on the effectiveness of clinical champions as an implementation strategy [2, 4, 8].

In order to know where clinical champions are in the ADKAR stages of change, there is a need for assessment tools, like the Practice Reflection Tool, that can be used for this identification and monitoring purpose. Tools like the Practice Reflection Tool that collect data on where clinical champions are within the change management process can greatly facilitate the tailoring of training to most effectively move champions through these processes. However, the Practice Reflection Tool has yet to be psychometrically tested beyond our pilot testing phase, which is a limitation for its use in other settings to test changes over time; this should be an area of future research. We have gone through three rounds of Practice Reflection Tool administration and have shared our findings with our partners at OPCC&CT who have used the tool’s results to inform their champion training activities. This work also demonstrates the value of working with implementation partners to develop evaluation tools to ensure that language and items are appropriate and actionable.

Understanding where clinical champions are along the stages of change will allow leaders of large system changes to better identify what is needed to prepare champions and tailor trainings to move them along the change process accordingly. While each change or transformation is unique, it may be useful to have a toolkit of resources specific for the clinical champion role, which to our knowledge does not exist. In the literature, there is little detail on training used to prepare clinical champions for their roles and this literature generally lacks a change management framing [3, 4, 8]. A toolkit of resources for clinical champions should be grounded in skills that have been identified as being essential for the clinical champion role, such as mentorship and communication, negotiation and advocacy, education and presentation, problem solving, and leadership [1, 6]. The development of an evidence-based, change management-grounded clinical champion toolkit is an important area for future research and practice to advance the clinical champion field.

One consideration when using the ADKAR-informed Conceptual Model to Prepare Clinical Champions for Facilitating Systems Change is that it assumes that strong organizational support for clinical champions and environments conducive to making change are already in place. The context in which clinical champions are working is essential to their success (e.g., supportive leadership is necessary to secure protected time for clinicians to carry out their clinical champion roles). Previous conceptual models have identified the importance of a variety of types of organizational support for clinical champions’ work, including having clear expectations, necessary time and resources, authority, and recognition/rewards [7]. In this way, the existence of organizational and leadership support may be considered a prerequisite to successful use of our conceptual model. However, use of this model may also strengthen learning health system culture (e.g., if clinical champions are successful at increasing uptake of a practice change within their clinic, leadership may be more supportive of their work and of implementation strategies more broadly), creating a reinforcing loop [33]. Future work could consider integration of our model with components of other types of implementation science models and frameworks like the Consolidated Framework for Implementation Research (CFIR) that include these “pre-requisite” factors at the “inner setting” level, like “culture” and “available resources” [34].

A second “assumption” when using this model is that the selected champions already have the position, trustworthiness, and respect from peers necessary for the clinical champion role. From the literature, these characteristics are all viewed as essential for clinical champions to be successful [1]. However, having these characteristics is likely necessary but not sufficient for enacting large-scale change within a healthcare system, as training to support movement through the multiple levels of the ADKAR change management process is still vital. It is also important to note that some characteristics of successful clinical champions can be taught (e.g., building coalitions, leadership qualities), while the ones mentioned above (e.g., position within the clinic, respect from peers) may be inherent or less “teachable” [7].

An area for future work is applying the model prospectively (since it was developed retrospectively), and refining the model based on these findings. Additionally, since the model was only developed based on data collected among WHICs in primary and mental healthcare within the VHA, the model should be applied in other settings/contexts in which clinical champions are working to either further refine it or identify adaptations that may be required if the model is applied outside of the WHIC setting. Given that the VHA had centralized support for national rollout of clinical champions, this setting and organizational culture may be distinct from other large healthcare systems and may require greater consideration of inner and outer setting determinants of implementation (as described above). Additionally, this model is focused on implementation within large healthcare systems and therefore may not be applicable to smaller-scale settings where clinical champions are being used.

## Conclusions

This work responds to a recognized need for guidance on training for clinical champions, particularly in large healthcare systems. Our data-driven, ADKAR-informed Conceptual Model to Prepare Clinical Champions for Facilitating Systems Change offers an approach that can be used within and outside of the VHA to improve the effectiveness of clinical champions, with the ultimate goal of increasing the use and spread of evidence-based practices across healthcare settings.

## Supplementary Information

Supplementary Material 1. SQUIRE checklist.

Supplementary Material 2.

## Data Availability

The datasets used and/or analysed during the current study are available from the corresponding author on reasonable request.

## Abbreviations

ADKAR Model: Awareness, Desire, Knowledge, Ability and Reinforcement Model
MH: Mental Health
OPCC&CT: Office of Patient Centered Care and Cultural Transformation
PC: Primary Care
VHA: Veterans Health Administration
WHIC: Whole Health Integration Champions
