# Supplementary material for: Preparing clinical champions for sustainable implementation of practice change within large healthcare systems
Source: Implement Sci Commun. 2026 Jan 31;7:43. doi: 10.1186/s43058-026-00873-7 (PMC12947433; doi:10.1186/s43058-026-00873-7)
Supplement: Supplementary file 2 — Supplementary Material 2. [file 43058_2026_873_MOESM2_ESM.docx]

**Supplementary Materials**

Supplementary Material 1. Example PRT Questions

Domain: Whole Health in Your Practice

| ***The following items are about your awareness and knowledge of Whole Health (WH) principles, approaches, and resources. For each statement, please select the option that best applies to you. Remember, there are no right or wrong answers.*** | | | | | |
| --- | --- | --- | --- | --- | --- |
| **How would you assess the strength of your knowledge in the following areas:** | 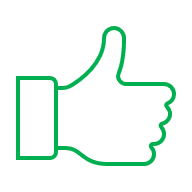  **Extensive**  **Knowledge** | 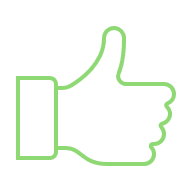 | 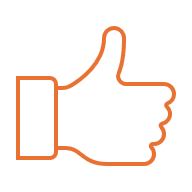  **Moderate knowledge** | 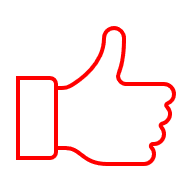 | 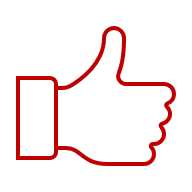  **Limited**  **Knowledge** |
| (a) Foundations of WH clinical care, such as person-centered communication, and health planning aligned with what matters most |  |  |  |  |  |
| (b) The Well-Being Signs, a 3-question measure that prompts Veterans to explore how satisfied, involved in, and functioning in activities that matter most to them |  |  |  |  |  |
| (c) How to use WH approaches in your clinical practice |  |  |  |  |  |
| (d) The role of WH Pathway services in a WH system of care |  |  |  |  |  |
| (e) The availability of Pathway services at your VA |  |  |  |  |  |
| (f) The evidence for complementary and integrative health (CIH) services that are required by Directive 1137 |  |  |  |  |  |
| (g) The availability of CIH services that are available at your VA |  |  |  |  |  |
| (h) The role of Health and Wellness Coaches |  |  |  |  |  |
| (i) The availability of Health and Wellness Coaches your VA? |  |  |  |  |  |
| (j) How to document Whole Health approaches in the medical record? |  |  |  |  |  |

Supplementary Material 2. Proportion of WHICs Reporting High Levels of Knowledge about Components of Whole Health Across PRT Rounds


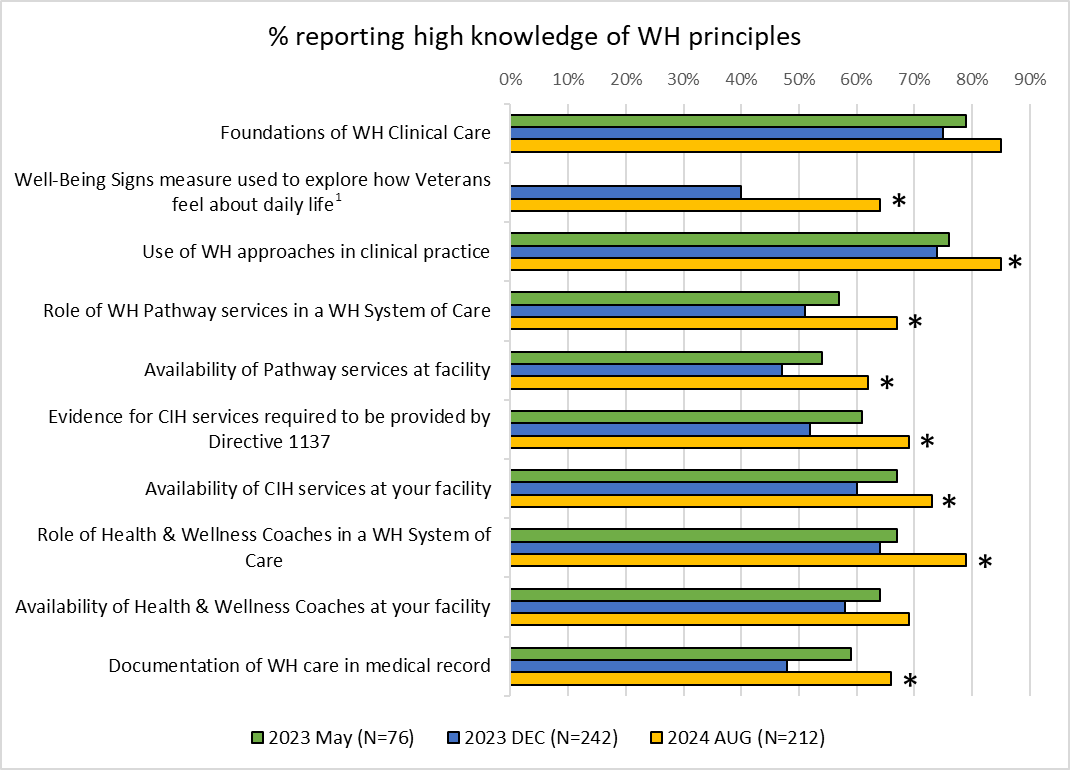
^1^The Well-Being Signs question was added following the 2023 administration

*Indicates significant difference (at p<0.01 level) between Dec 2023 and Aug 2024

Supplementary Material 3. Proportion of WHICs Reporting High Levels of Knowledge of the WHIC Role Across PRT Rounds


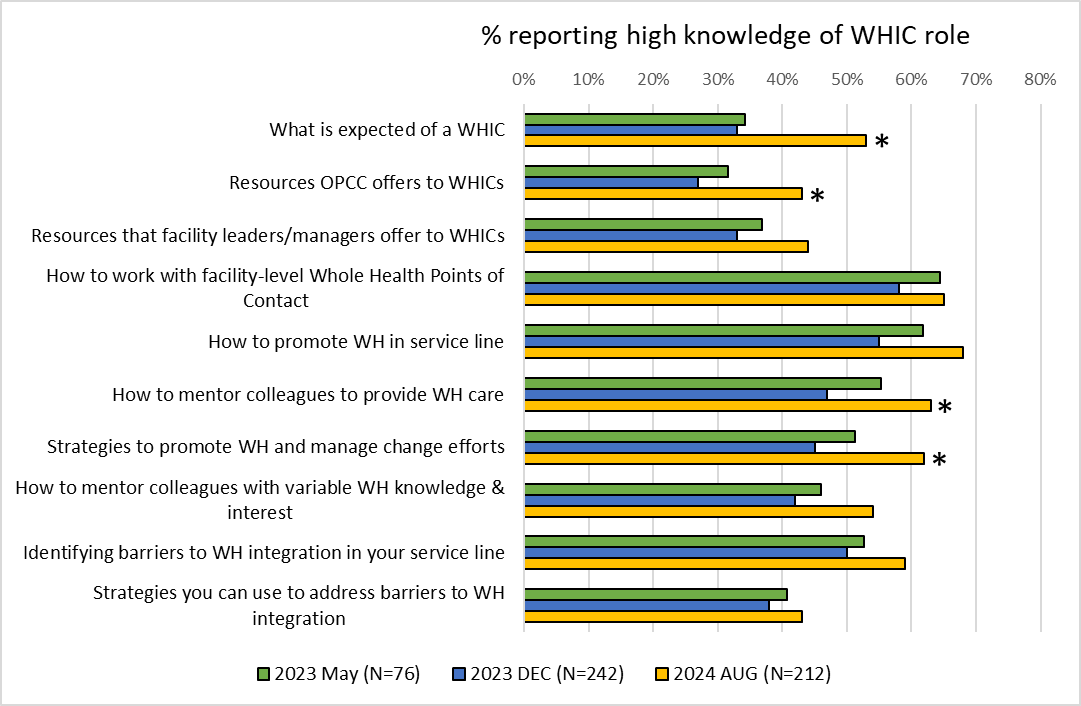
*Indicates significant difference (at p<0.01 level) between Dec 2023 and Aug 2024
